# Apical sodium-dependent bile acid transporter inhibition with volixibat improves metabolic aspects and components of non-alcoholic steatohepatitis in Ldlr-/-.Leiden mice

**DOI:** 10.1371/journal.pone.0218459

**Published:** 2019-06-24

**Authors:** Kanita Salic, Robert Kleemann, Cynthia Wilkins-Port, John McNulty, Lars Verschuren, Melissa Palmer

**Affiliations:** 1 TNO, Department of Metabolic Health Research, Leiden, Netherlands; 2 Shire LLC, now part of Takeda, Cambridge, Massachusetts, United States of America; 3 TNO, Department of Microbiology and Systems Biology, Zeist, Netherlands; Universidade do Estado do Rio de Janeiro, BRAZIL

## Abstract

Interruption of bile acid recirculation through inhibition of the apical sodium-dependent bile acid transporter (ASBT) is a promising strategy to alleviate hepatic cholesterol accumulation in non-alcoholic steatohepatitis (NASH), and improve the metabolic aspects of the disease. Potential disease-attenuating effects of the ASBT inhibitor volixibat (5, 15, and 30 mg/kg) were investigated in high-fat diet (HFD)-fed Ldlr-/-.Leiden mice over 24 weeks. Plasma and fecal bile acid levels, plasma insulin, lipids, and liver enzymes were monitored. Final analyses included liver histology, intrahepatic lipids, mesenteric white adipose tissue mass, and liver gene profiling. Consistent with its mechanism of action, volixibat significantly increased the total amount of bile acid in feces. At the highest dose, volixibat significantly attenuated the HFD-induced increase in hepatocyte hypertrophy, hepatic triglyceride and cholesteryl ester levels, and mesenteric white adipose tissue deposition. Non-alcoholic fatty liver disease activity score (NAS) was significantly lower in volixibat-treated mice than in the HFD controls. Gene profiling showed that volixibat reversed the inhibitory effect of the HFD on metabolic master regulators, including peroxisome proliferator-activated receptor-γ coactivator-1β, insulin receptor, and sterol regulatory element-binding transcription factor 2. Volixibat may have beneficial effects on physiological and metabolic aspects of NASH pathophysiology.

## Introduction

Over the past two decades, rising rates of obesity, type 2 diabetes mellitus (T2DM), and metabolic syndrome have been accompanied by an increasing prevalence of non-alcoholic steatohepatitis (NASH).[1, 2] NASH is a severe and often progressive form of non-alcoholic fatty liver disease (NAFLD) for which there is no approved pharmacological therapy.[3, 4] The US Food and Drug Administration has designated a fast-track approval process for numerous drugs in development for the treatment of NASH, underscoring the urgent need for pharmacological treatment options.[5]

Histologically, NASH is characterized by hepatic steatosis, lobular inflammation, and hepatocyte injury (ballooning), leading to cell death, which can occur with or without fibrosis.[6] NASH is typically asymptomatic and a liver biopsy is required for diagnosis, making early detection difficult.[6, 7] NASH can lead to cirrhosis, hepatocellular carcinoma (HCC),[8] and liver failure,[9–12] and is the leading reason for liver transplantation in US adults under the age of 50 years.[13]

The pathophysiology of NASH is both complex and multifactorial and has not been definitively identified. It most likely includes combinations of genetic, environmental, and gut microbial factors, in addition to abnormal metabolism of bile acids, lipids (including cholesterol), and insulin resistance (IR).[14, 15] Increased synthesis coupled with reduced hepatic excretion of cholesterol results in the accumulation of free cholesterol,[16] which is associated with hepatocyte injury, predominantly through disruption of mitochondrial function [17] and promotion of oxidative stress.[18]

NASH is also associated with increased serum insulin and triglyceride concentrations, accompanied by elevated levels of very-low-density lipoprotein cholesterol and reduced levels of high-density lipoprotein cholesterol.[18, 19] IR and hyperlipidemia are common in patients with NASH, as are metabolic disorders and visceral obesity.[2] Expansion of white adipose tissue may contribute to disease progression through the production of adipokines, inflammatory cytokines and lipids.[20, 21]

**Fig 1:**
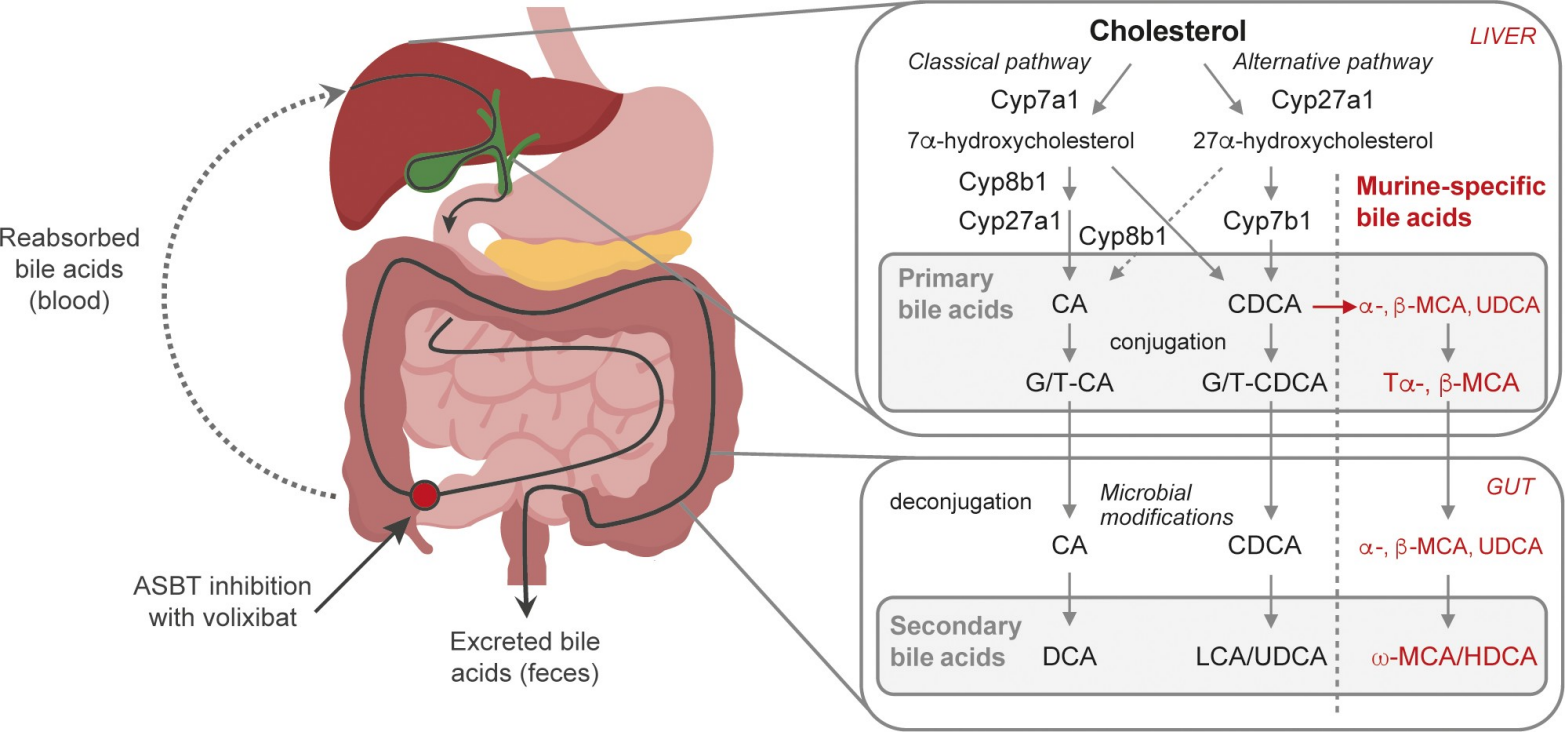
Bile acid synthesis and recirculation. Primary bile acids are synthesized from cholesterol in the liver, via either the classical pathway, which produces CA, or the alternative pathway, which produces CDCA, or, in mice only, α- or β-MCA.[27, 30] Bile acids are conjugated with glycine or taurine (predominantly taurine in mice) before being released into the bile.[30] In the gut, bile acids are deconjugated and metabolized into secondary bile acids [27, 30, 31] (UDCA is considered a secondary bile acid in humans [32] but a primary bile acid in mice).[26] About 95% of bile acids are reabsorbed from the gut and transported back to the liver via the hepatic portal vein, and the remainder are excreted.[27, 31, 33] The hydrophilicity of the common free and conjugated bile salts decreases in the order UDCA > CA > CDCA > DCA) > LCA, and taurine-conjugated > glycine-conjugated > free species.[34] ASBT inhibition with volixibat blocks the reabsorption of bile acids and increases their excretion, stimulating the liver to synthesize more bile acids from cholesterol.[35–37] ASBT: apical sodium-dependent bile acid transporter, CA: cholic acid, CDCA: chenodeoxycholic acid, Cyp: cytochrome P450 family, DCA: deoxycholic acid, G: glycine, HDCA: hyodeoxycholic acid, LCA: lithocholic acid, MCA: muricholic acid, T: taurine, UDCA: ursodeoxycholic acid.

Serum bile acid levels are often elevated in individuals with NASH, reaching concentrations up to twofold higher than normal.[22] A rise in bile acid levels can trigger inflammatory responses, oxidative stress, and necrotic cell death pathways, which may also contribute to liver injury.[23–25] Primary bile acids are synthesized from cholesterol in the liver and transported in bile to the gut, where they can be metabolized into secondary bile acids by bacteria (Fig 1).[19, 26, 27] Secondary bile acids, for example lithocholic acid (LCA) and deoxycholic acid (DCA), are more hydrophobic [25–28] and may trigger inflammatory responses, increase the production of reactive oxygen species, and induce necrotic cell death pathways.[25, 28, 29]

Despite the rising prevalence of NASH, recommended treatment options are limited to dietary changes and exercise.[3, 38, 39] An abundance of potential pharmacological strategies are currently in development.[38] One approach is to interrupt the enterohepatic recirculation of bile acids to the liver by inhibiting the apical sodium-dependent bile acid transporter (ASBT).[40] The ASBT is a transmembrane protein localized on the luminal surface of ileal enterocytes. Approximately 95% of the bile acid pool is reabsorbed in the terminal ileum and then returned to the liver for reuse, while the remainder leaves the body via the feces (Fig 1).[27, 33]

Interrupting bile acid recirculation by inhibiting the ASBT typically increases the bile acid content in feces,[35, 40, 41] and is thought to stimulate the liver to synthesize new bile acids from hepatic cholesterol in order to maintain bile acid homeostasis.[35–37] Indeed, in mice, ASBT inhibition increased hepatic expression of cholesterol 7α-hydroxylase (Cyp7a1),[35] a rate-limiting enzyme in bile acid synthesis.[42, 43] Similarly, in clinical studies, ASBT inhibition was associated with an increase in serum levels of 7α-hydroxy-4-cholesten-3-one (C4), a biomarker of bile acid synthesis downstream of Cyp7a1.[36] ASBT inhibition may provide therapeutic benefit for patients with NASH by promoting the use of cholesterol as a substrate for bile acid synthesis, thereby reducing systemic and hepatic cholesterol levels and potentially attenuating the metabolic stress and liver injury associated with hepatic cholesterol accumulation.[36, 40, 41]

Volixibat potassium (SHP626; formerly LUM002; hereafter referred to as volixibat) is a potent and minimally absorbed ASBT inhibitor.[40, 44] The current mouse study was performed in parallel with a phase 2 clinical study in patients with NASH (ClinicalTrials.gov Identifier: NCT02787304) in order to elucidate the effects of ASBT inhibition on metabolic aspects and components of NAFLD/NASH, including putative effects on metabolically critical pathways and transcription factors (e.g. via transcriptomics-based upstream regulator analysis of farnesoid X receptor [FXR] target genes), important information not previously reported. To do so, Ldlr-/-.Leiden mice were fed a high-fat diet (HFD) to induce obesity, steatosis, inflammation, IR, and hyperlipidemia (i.e. a phenotype that resembles human NASH).[40] Disease induction was limited to 24 weeks to optimally establish metabolic dysfunction in NASH without histological manifestation of fibrosis. Volixibat was administered at three doses allowing the study of potential dose-dependent effects of ASBT inhibition on metabolic pathogenic processes and NASH pathology.

## Materials and methods

### Experimental design

**Fig 2:**
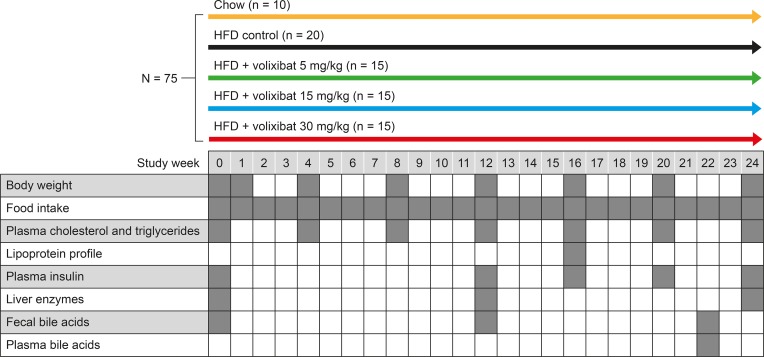
Study design and schedule of assessments during the study. An oral glucose tolerance test was performed in week 18. HFD: high-fat diet.

Mice (*N* = 75, between 16–18 weeks old) were matched by body weight and plasma cholesterol, triglyceride, and blood glucose levels, divided into five study groups, and maintained on either a chow diet (*n* = 10), a HFD (*n* = 20), or a HFD supplemented with volixibat 5, 15, or 30 mg/kg (*n* = 15 in each dose group) for 24 weeks (Fig 2).

Food intake and body weight were monitored throughout the study to ensure accurate volixibat dosing. The schedule of assessments over the 24 weeks is shown in Fig 2, and included measurements of plasma cholesterol triglycerides, insulin, and liver enzymes, and determination of the lipoprotein profile. Bile acid species were measured in plasma and feces. Plasma samples (collected at weeks 0, 4, 8, 12, 16, 20, and 24) were obtained via a tail bleed after 5 hours of fasting (Fig 2). The mice were sacrificed at week 24 and the livers were isolated for histological evaluation of NASH [45] and liver lipid analysis. White adipose tissue depots were also collected and weighed. Gene expression analysis was carried out on liver tissue from eight mice in each group, selected as being representative of the group based on mean values of histological NASH parameters. All analyses were performed blindly.

### Animal husbandry and ethics

Male Ldlr-/-.Leiden mice were characterized [46, 47] and bred by TNO Metabolic Health Research at facilities of InnoSer Laboratories (Leiden, The Netherlands). Mice were housed in Makrolon cages (two to four mice per cage) at approximately 21°C, with a 12-hour daily light cycle and relative humidity of 50–60%. The mice were supplied with food and tap water *ad libitum*, and were fed either standard chow (Ssniff R/MH, Soest, Germany) or a NASH-inducing HFD (containing 45% kcal fat from lard, 35% kcal from carbohydrates [primarily sucrose] and 20% kcal from protein; D12451, Research Diets, New Brunswick, NJ) as described previously.[46–48] The mice were sacrificed by carbon dioxide asphyxiation.

All experiments were performed in concordance with the Netherlands law on animal experiments and were approved by an independent committee on animal care and experimentation (Dierexperimentencommissie Zeist, Netherlands; approval number 3682/TNO-130), in a facility accredited by the Association for Assessment and Accreditation of Laboratory Animal Care.

### Determination of bile acids in feces

Feces from each cage were collected over a 2-day period during weeks 0, 12, and 22. Samples of dried feces (5 mg) were incubated in 1 mL of alkaline methanol (3:1 v/v) for 2 hours at 80°C with nor-hyodeoxycholate as an internal standard, as described previously.[49] The samples were diluted in distilled water, mixed, and centrifuged. The supernatant was then applied to a Sep-Pak C18 solid-phase extraction cartridge (Waters Corporation, Wexford, Ireland). Bile acids were eluted with 100% methanol and then desiccated. Bile acids were derivatized by incubation with 50 μL of trifluoroacetic anhydride and 30 μL of 1,1,1,3,3,3-hexafluoro-2-propanol for 1 hour at 60°C. Separation of bile acid species was achieved using a 25 m × 0.25 mm capillary gas chromatography column (CP-Sil 5B, Agilent, Santa Clara, California, USA) in a Scion 436-GC gas chromatography system (Scion Instruments, Livingstone, UK) equipped with a flame ionization detector. The injector and flame ionization detector were maintained at 280°C. The column temperature was programmed to change from 230°C to 280°C. Quantitation was based on the area ratio of each individual bile acid to the internal standard. In total, seven bile acid species were measured; cholic acid (CA), alpha-muricholic acid (α-MCA), beta-muricholic acid (β-MCA), deoxycholic acid (DCA), lithocholic acid (LCA), omega-muricholic acid (ω-MCA) and hyo-DCA/urso-DCA (HDCA/UDCA). The sum of these seven bile acids was used to calculate the total amount of bile acid in feces.

### Determination of bile acids in plasma

Bile acids were determined using high-performance liquid chromatography (HPLC) with mass spectrometry (MS). Calibration standards were prepared for individual deuterated bile acids at concentrations of 0.005–40 μM. Samples were prepared as previously described.[50] HPLC–MS was performed using a Dionex UltiMate 3000 HPLC system coupled with a Q Exactive mass spectrometer (Thermo Fisher Scientific, Breda, the Netherlands) and an Acquity BEH C18 column (2.1 mm × 50 mm, 1.7 μm, Waters, Etten-Leur, the Netherlands) at 40°C. The ratio of mobile phase A (1 mM ammonium formate in water [pH 4.4]) to mobile phase B (acetonitrile:water [95:5 v/v] containing 1 mM ammonium formate) was varied over 14 minutes, with a flow rate of 600 μL/minute. The injection volume was 3 μL and the autosampler temperature was 20°C. The mass spectrometer, equipped with a heated electrospray ionization source, was operated in negative mode and full-scan spectra were recorded. The spray voltage was 3 kV, and capillary and probe heater temperatures were 350°C and 320°C, respectively. Nitrogen was used as the sheath and auxiliary gas, set at 60 and 20 (arbitrary units), respectively. The resolution was 100,000 at m/z 200. The data were analyzed using LCQUAN software (Thermo Fisher Scientific, Inc., Waltham, Massachusetts, USA).

### Blood and plasma analysis

Plasma cholesterol and triglyceride levels were measured using commercial enzymatic kits (Roche Diagnostics, Basel, Switzerland). Insulin was measured using an ultrasensitive mouse insulin enzyme-linked immunosorbent assay kit (Crystal Chem, Zaandam, the Netherlands). Plasma alanine aminotransferase (ALT) and aspartate aminotransferase (AST) levels were measured using a spectrophotometric activity assay (Reflotron system, Boehringer Ingelheim, Mannheim, Germany). For lipoprotein profiling, 50 μL of plasma collected and pooled from the mice in each treatment group at week 16 were applied to a 25 mL Superose 6B column (Pharmacia AB, Uppsala, Sweden) connected to an ÄKTA fast protein liquid chromatography system (Amersham Pharmacia Biotech, Amersham, UK [now GE Healthcare]), and separated at a constant flow rate of 50 μL/minute with phosphate-buffered saline (pH 7.4). The eluent was collected in 50 μL fractions (24 fractions in total), and cholesterol fractions were measured using an enzymatic kit (Roche Diagnostics).

### Liver tissue analysis

Paraffin-embedded cross sections were stained with hematoxylin and eosin, and scored by a pathologist using a grading system adapted for NASH.[51] The level of steatosis was determined over two liver cross sections per mouse, and was expressed as a percentage of the liver area analyzed. Hepatic inflammation was assessed by counting the number of inflammatory foci per field at 100 × magnification in five non-overlapping fields per specimen. Histological sections were also assessed using the NAFLD activity score (NAS),[52] defined as the sum of the scores for steatosis (0–3), lobular inflammation (0–3), and liver injury (ballooning; 0–2). Lipids were extracted from liver tissue using the Bligh and Dyer method [53] and were quantified as previously described.[54] Hydroxyproline was measured in freshly prepared liver homogenates using a total collagen assay (Quickzyme, Leiden, the Netherlands) relative to a collagen standard following the manufacturer’s instructions.[40]

### Gene profiling

Total RNA from 8 animals per group was extracted from snap-frozen liver tissue using the Ambion RNAqueous total RNA isolation kit (Thermo Fisher Scientific, Inc.). The RNA concentration was measured using a NanoDrop 1000 spectrophotometer (Isogen Life Science, De Meern, Netherlands). RNA quality was assessed using the 2100 Bioanalyzer (Agilent Technologies, Amstelveen, Netherlands). Samples were prepared in accordance with the manufacturer’s protocol using the NEBNext Ultra Directional RNA Library Prep Kit for Illumina (New England Biolabs, Ipswich, Massachusetts, USA). mRNA was isolated from total RNA using the NEBNext Poly(A) mRNA magnetic isolation module (New England Biolabs). cDNA synthesis was performed and used for ligation with the sequencing adapters and polymerase chain reaction (PCR) amplification of the resulting product. The quality and yield of the PCR products were consistent with the expected size distribution (300–800 base pairs). Clustering and DNA sequencing were performed by GenomeScan BV (Leiden, Netherlands) using a NextSeq 500 sequencer (Illumina, San Diego, California, USA) with a single-read 75-cycle sequencing protocol with at least 15 million reads per sample. The sequences were directly aligned with the mouse reference genome (GRCm38p4) using the Start 2.5 algorithm with default settings. DESeq [55] was used to evaluate differential expression between treatment groups. Differentially expressed genes (DEGs; α = 0.01) were used as an input for pathway analysis using the Ingenuity Pathway Analysis suite (Qiagen, Hilden, Germany). Gene expression data is accessible at Gene Expression Omnibus (https://www.ncbi.nlm.nih.gov/geo/) under GSE129306. The effects of the treatment were analyzed by gene enrichment analysis across pathways and biological processes using Ingenuity Pathway Analysis suite (IPA; www.ingenuity.com) and the upstream regular analysis tool of IPA was used to assess the activity of transcription factors as well as other upstream regulators essentially as reported.[56] Gene expression data of all known target genes of a transcription factor were used to predict activation or deactivation of the upstream regulator in question. A negative z-score of less than -2 indicates significantly reduced transcriptional activity based on the direction of gene expression changes of target genes. Conversely, a positive z-score of greater than 2 indicates significant activation of the upstream regulator essentially as reported in translational studies.[47]

### Statistical analysis

All statistical comparisons were against the HFD control group. For normally distributed data with equal variances, a one-way analysis of variance with Dunnett’s *post hoc* test was used (α = 0.05). For data sets that were not normally distributed or did not have equal variances, a Kruskal–Wallis test was used. When the result of the Kruskal–Wallis test indicated a significant difference (*P* < 0.05), a Mann–Whitney U test was used to compare independent samples. An overview of the statistical tests that were used for each of the figures is shown in S1 Table. For the pathway analysis of differentially expressed genes, *P* values were based on Fisher’s exact test (α = 0.01). All *P* values reported in the study are considered nominal because comparisons were not corrected for multiplicity.

## Results

### Volixibat increased the total amount of bile acid in feces

**Fig 3:**
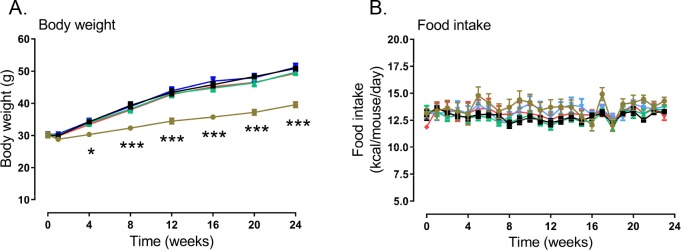
Body weight and food intake throughout the study. (A) body weight and (B) food intake. Error bars show standard error of the mean. *P < 0.05; ***P < 0.001 versus the HFD control group. Chow group, *n* = 10; HFD control group, *n* = 20; HFD + volixibat 5 mg/kg, *n* = 15; HFD + volixibat 15 mg/kg, *n* = 15; HFD + volixibat, *n* = 15.

During the study, no signs of animal discomfort or diarrhea were observed in mice treated with volixibat. The body weight and food intake of volixibat-treated mice (Fig 3A and 3B) did not differ from the HFD control mice and there was no dropout of animals during the study.

**Fig 4:**
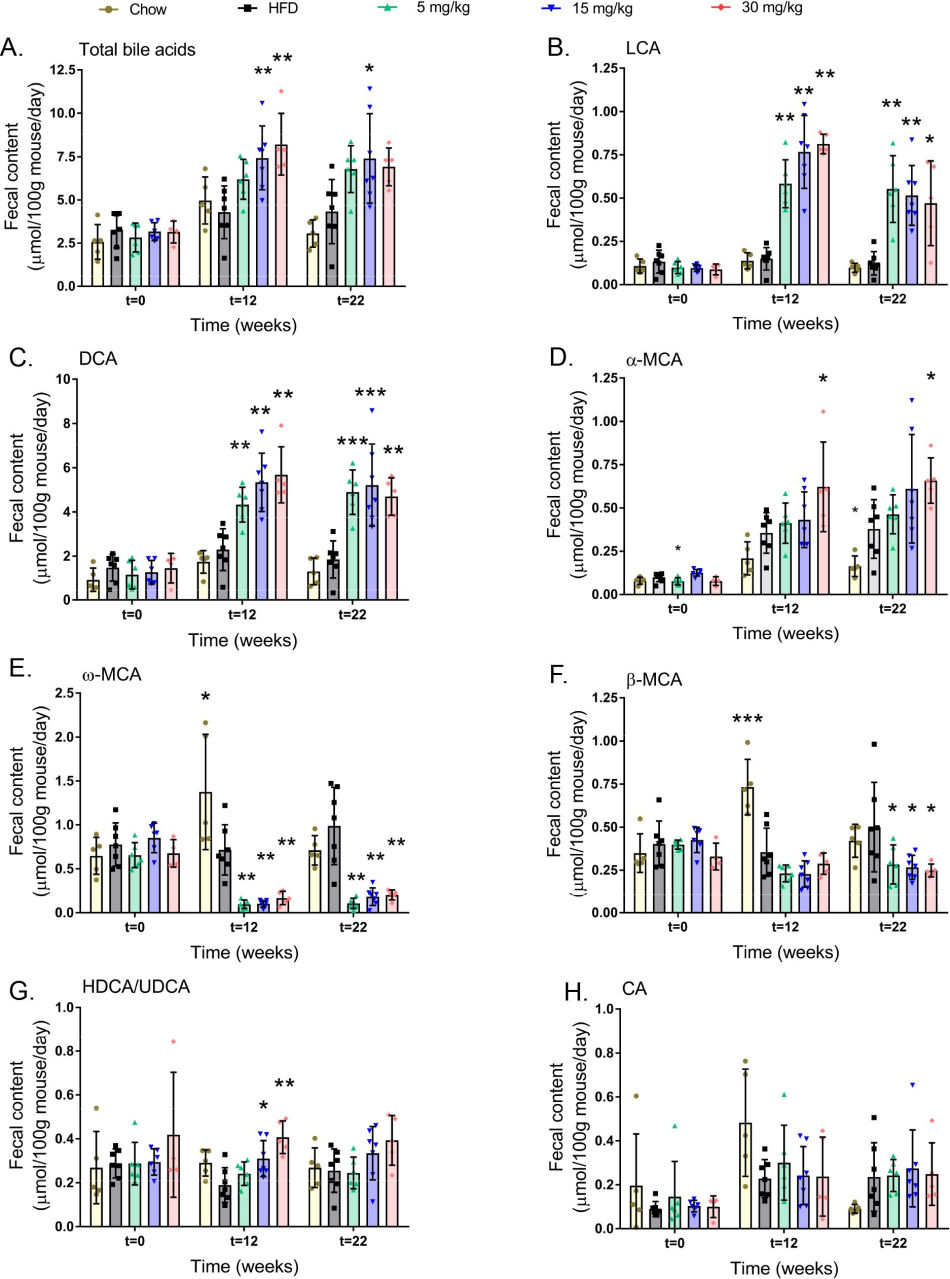
Mean fecal bile acid content at baseline, 12 weeks, and 22 weeks. (A) total bile acids, (B) LCA, (C) DCA, (D) α-MCA, (E) ω-MCA, (F) β-MCA, (G) HDCA/UDCA and (H) CA. Horizontal lines indicate mean values. Error bars show standard deviation. Fecal bile acid content was measured for mice in each cage over a 2-day period at 0, 12, and 22 weeks. *P < 0.05; **P < 0.01; ***P < 0.001 versus the HFD control group. CA: cholic acid, DCA: deoxycholic acid, HDCA/UDCA: hyodeoxycholic acid/ursodeoxycholic acid, HFD: high-fat diet, LCA: lithocholic acid, MCA: muricholic acid. All data points shown.

Volixibat treatment was associated with a dose-dependent increase in the total amount of bile acid in feces at week 12. The biggest modulation was observed in the 30 mg/kg dose group with levels increasing from 3.15 ± 0.64 μmol/100 g mouse/day at baseline to 8.21± 1.78 μmol/100 g mouse/day at week 12. This effect was sustained, with similar levels observed at weeks 22 at all doses. Total fecal bile acid levels were similar for the chow and HFD control groups, and remained similar to baseline levels throughout the study (Fig 4A).

### Volixibat modulates the fecal content of specific bile acids

Volixibat treatment resulted in profound changes in fecal concentrations of specific bile acids (Fig 4B–4H). Fecal concentrations of LCA and DCA were comparable at the start of the study but increased significantly in the volixibat groups (all doses) compared to the HFD control group in both week 12 and 22 (Fig 4B and 4C, respectively). Fecal DCA concentrations were much greater in magnitude than those of any other bile acid species. In addition, the fecal concentration of α-muricholic acid (MCA) increased significantly in the 30 mg/kg dose group relative to the HFD control group at weeks 12 and 22 (Fig 4D).

In contrast, fecal ω-MCA levels decreased substantially in all volixibat groups at weeks 12 and 22 (Fig 4E; *P* < 0.01). Also, β-MCA concentration declined with all volixibat doses at week 22 (Fig 4F; *P* < 0.05). Fecal hyo-DCA/urso-DCA (HDCA/UDCA) levels increased in week 12 in mid (*P* < 0.05) and high dose *(P* < 0.01) groups, while no significant effects were observed in week 22 (Fig 4G). Fecal cholic acid (CA) levels were comparable between the volixibat and the HFD groups at all three time-points (Fig 4H).

### Volixibat modulates specific plasma bile acid species

**Fig 5:**
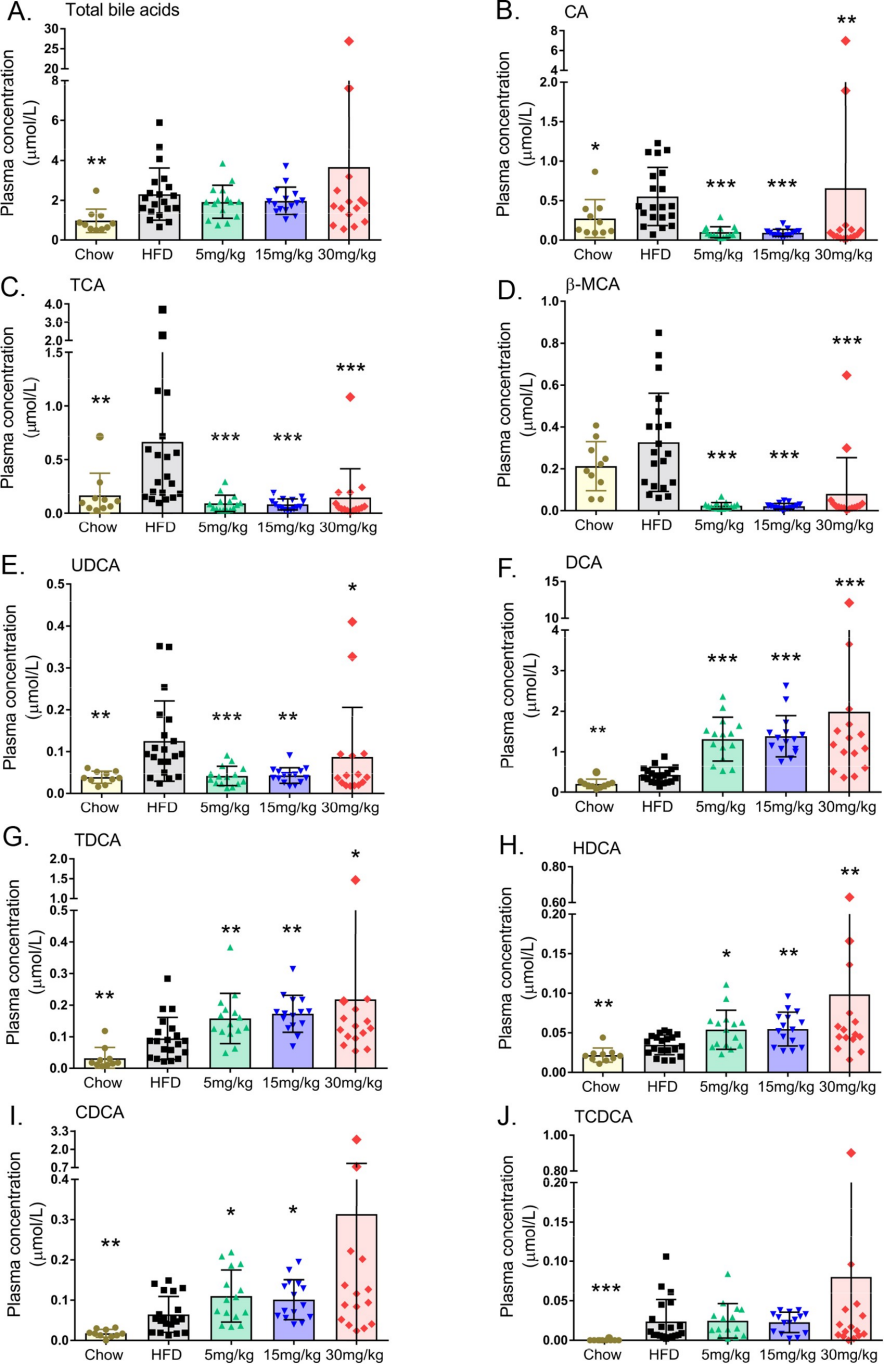
Mean plasma bile acid levels at 22 weeks. (A) total bile acids, (B) CA, (C) TCA, (D) β-MCA, (E) UDCA, (F) DCA, (G) TDCA, (H) HDCA, (I) CDCA, and (J) TCDCA. Dose groups indicate HFD + volixibat dose. Horizontal lines indicate mean values. Error bars show standard deviation. *P < 0.05; **P < 0.01; ***P < 0.001 versus the HFD control group. CA: cholic acid, CDCA: chenodeoxycholic acid, DCA: deoxycholic acid, HDCA: hyodeoxycholic acid, HFD: high-fat diet, MCA: muricholic acid, TCA: taurine-conjugated cholic acid, TCDCA: taurine-conjugated chenodeoxycholic acid, TDCA: taurine-conjugated deoxycholic acid, UDCA: ursodeoxycholic acid. All data points shown.

Compared with the chow diet, the HFD resulted in a significant increase in total plasma bile acid concentrations (Fig 5A). Although volixibat treatment was not associated with changes in total bile acid levels, volixibat modified the levels of specific circulating bile acids.

Volixibat significantly attenuated the HFD-induced increase in plasma CA, taurine-conjugated CA (TCA), β-MCA, and UDCA levels, all of which were reduced to similar values to those observed in the chow group (Fig 5B–5E). In contrast, DCA, taurine-conjugated DCA (TDCA), HDCA, and chenodeoxycholic acid (CDCA) levels were all significantly higher in the volixibat-treated groups than in the HFD control group (*P* < 0.05 for all, except for CDCA in the 30 mg/kg group [*P* = 0.067]; Fig 5F–5I). Plasma taurine-conjugated CDCA (TCDCA) levels in the volixibat-treated group were similar to those of the HFD control group (Fig 5J). Plasma LCA levels were below detection limit in all groups.

### Volixibat attenuates metabolic risk factors and liver enzyme ALT

**Fig 6:**
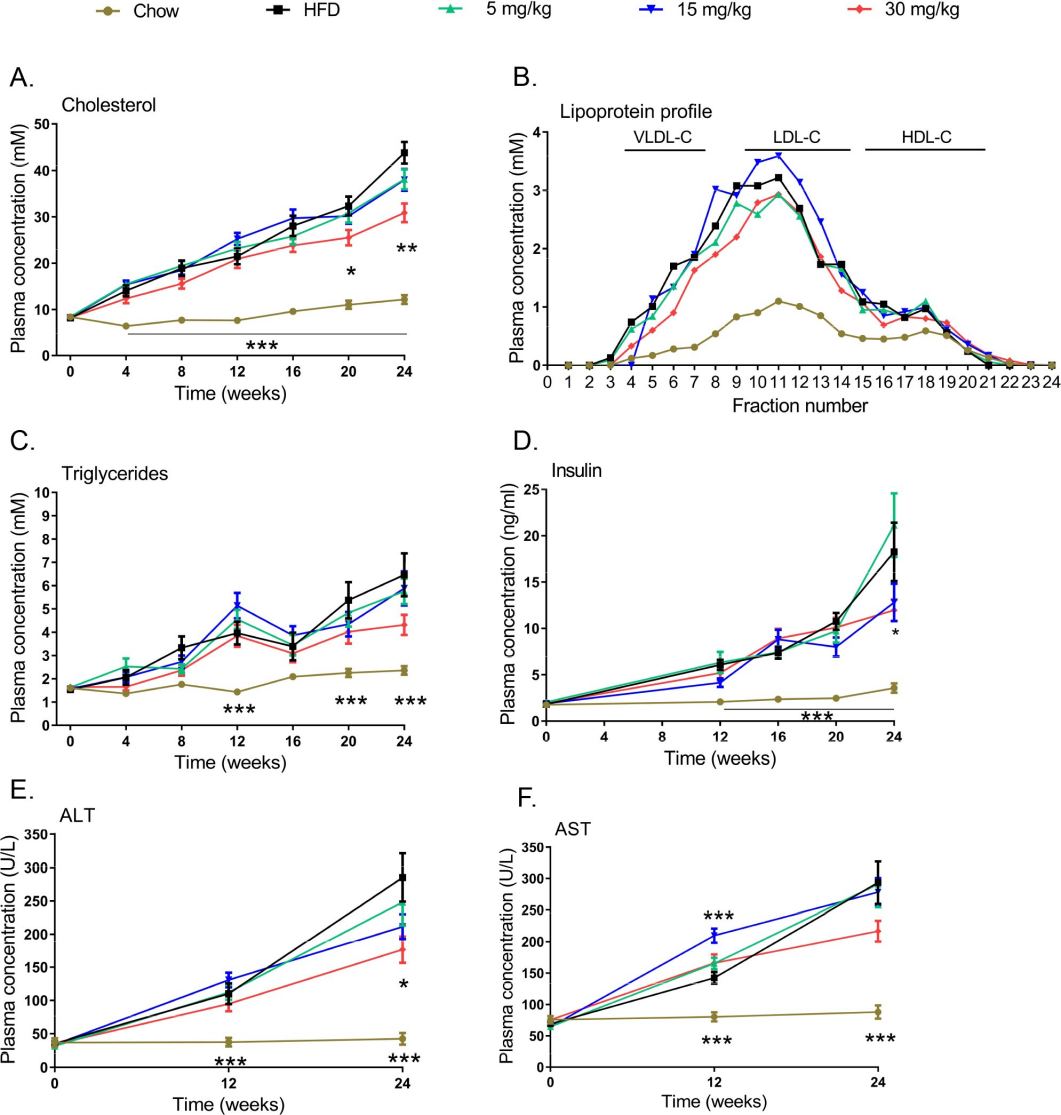
Effects of volixibat treatment on plasma lipids, insulin and liver enzymes throughout the study. (A) cholesterol, (B) lipoprotein profiles, (C) triglycerides, (D) insulin, (E) ALT and (F) AST. Error bars show standard error of the mean. Lipoprotein analysis was performed using plasma pooled from all mice in each group (at week 16). Data are absolute values and represent cholesterol concentrations in eluted fractions. *P < 0.05; **P < 0.01; ***P < 0.001 versus the HFD control group. ALT: alanine aminotransferase, AST: aspartate aminotransferase, HDL-C: high-density lipoprotein cholesterol, HFD: high-fat diet, LDL-C: low-density lipoprotein cholesterol, VLDL-C: very-low-density lipoprotein cholesterol. Chow group, *n* = 10; HFD control group, *n* = 20; HFD + volixibat 5 mg/kg, *n* = 15; HFD + volixibat 15 mg/kg, *n* = 15; HFD + volixibat, *n* = 15.

The HFD resulted in a steady increase in plasma cholesterol, lipoprotein profiles, triglyceride levels, insulin, ALT and AST, which were all consistently higher than those in the chow-fed mice during the study (*P* < 0.001 for all except plasma triglycerides, which were not significantly elevated at week 16; Fig 6).

At the highest dose, volixibat significantly attenuated the HFD-induced increase in plasma cholesterol (from week 20 onwards) (Fig 6A), while lipoprotein profiles and plasma triglycerides were only slightly affected (Fig 6B and 6C).

Volixibat significantly reduced plasma insulin levels at week 24 (Fig 6D), while glucose levels were comparable to the HFD group. At week 18, an oral glucose tolerance test was performed to assess glucose metabolism. There was no difference between the HFD group and the volixibat treated groups. The average area under the curve (AUC) was 569 ± 247 for HFD, 536 ± 408 for 5 mg/kg group, 460 ± 186 for 15 mg/kg group and 598 ± 454 for 30 mg/kg group.

Plasma ALT levels were significantly lower in the highest dose group than in the HFD control group at week 24 (Fig 6E; *P* < 0.05); AST levels were also lower in the highest dose group but not significant different to the HFD control group (Fig 6F).

### Volixibat ameliorates hepatic lipids and NAFLD activity score

**Fig 7:**
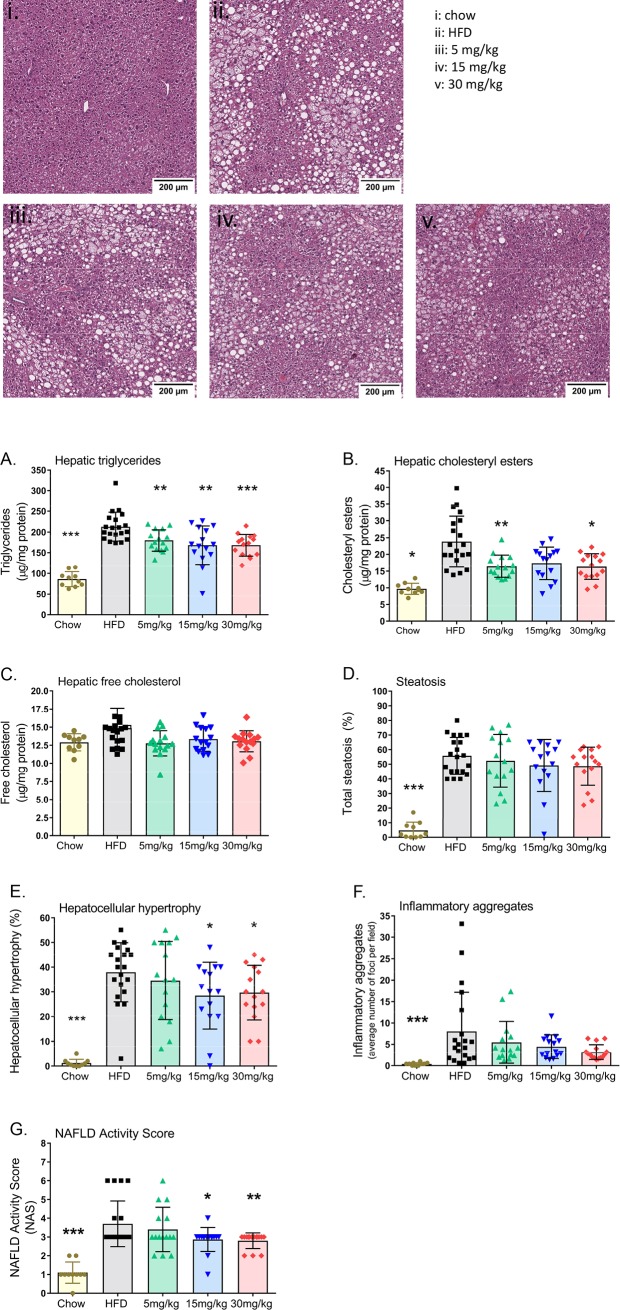
Effects of volixibat on liver pathology at the end of the study. Representative hematoxylin and eosin photomicrographs of (i) chow, (ii) HFD, (iii) 5 mg/kg volixibat, (iv) 15 mg/kg volixibat (v) 30 mg/kg volixibat. (A) hepatic triglycerides, (B) hepatic cholesteryl esters, (C) hepatic free cholesterol, (D) steatosis, (E) hepatocyte hypertrophy, (F) inflammatory aggregates and (G) NAFLD activity score. Dose groups indicate HFD + volixibat dose. Horizontal lines indicate mean values. Error bars show standard deviation. Liver histology and liver lipids were assessed after sacrifice of mice at the end of the study. *P < 0.05; **P < 0.01; ***P < 0.001 versus the HFD control group. HFD: high-fat diet, NAFLD: non-alcoholic fatty liver disease. All data points shown.

Mice in the HFD control group had significantly higher levels of hepatic triglycerides than the chow group. Volixibat treatment significantly attenuated the HFD-induced increase in hepatic triglyceride and cholesteryl ester levels (Fig 7A and 7B; *P* < 0.05). Although there were no significant differences in hepatic free cholesterol between the groups, volixibat-treated mice had similar cholesterol levels to those in the chow group (Fig 7C).

Histopathological analysis of livers revealed significant HFD-induced increases in total steatosis, hepatocellular hypertrophy, lobular inflammation and NAS (Fig 7D–7G). Volixibat treatment with 15 mg/kg and 30 mg/kg significantly reduced HFD-induced hypertrophy (*P* < 0.05) and NAS (*P* < 0.05). Fibrosis was not established at 24 weeks of HFD treatment. Biochemical analysis of hepatic hydroxyproline levels showed no effect of volixibat treatment on collagen content (Figure A in S1 Fig). Consistent with this, the HFD was associated with increased expression of collagen genes (Figure B in S1 Fig). Processes affected by the HFD included ‘Early signaling events in hepatic stellate cells’ and ‘Signaling events in activated HSC’ (Figure C in S1 Fig). Volixibat treatment had no effect on these genes or processes (Figures D–F in S1 Fig).

### Volixibat attenuates mesenteric white adipose tissue weight and modifies key regulators in energy metabolism

**Fig 8:**
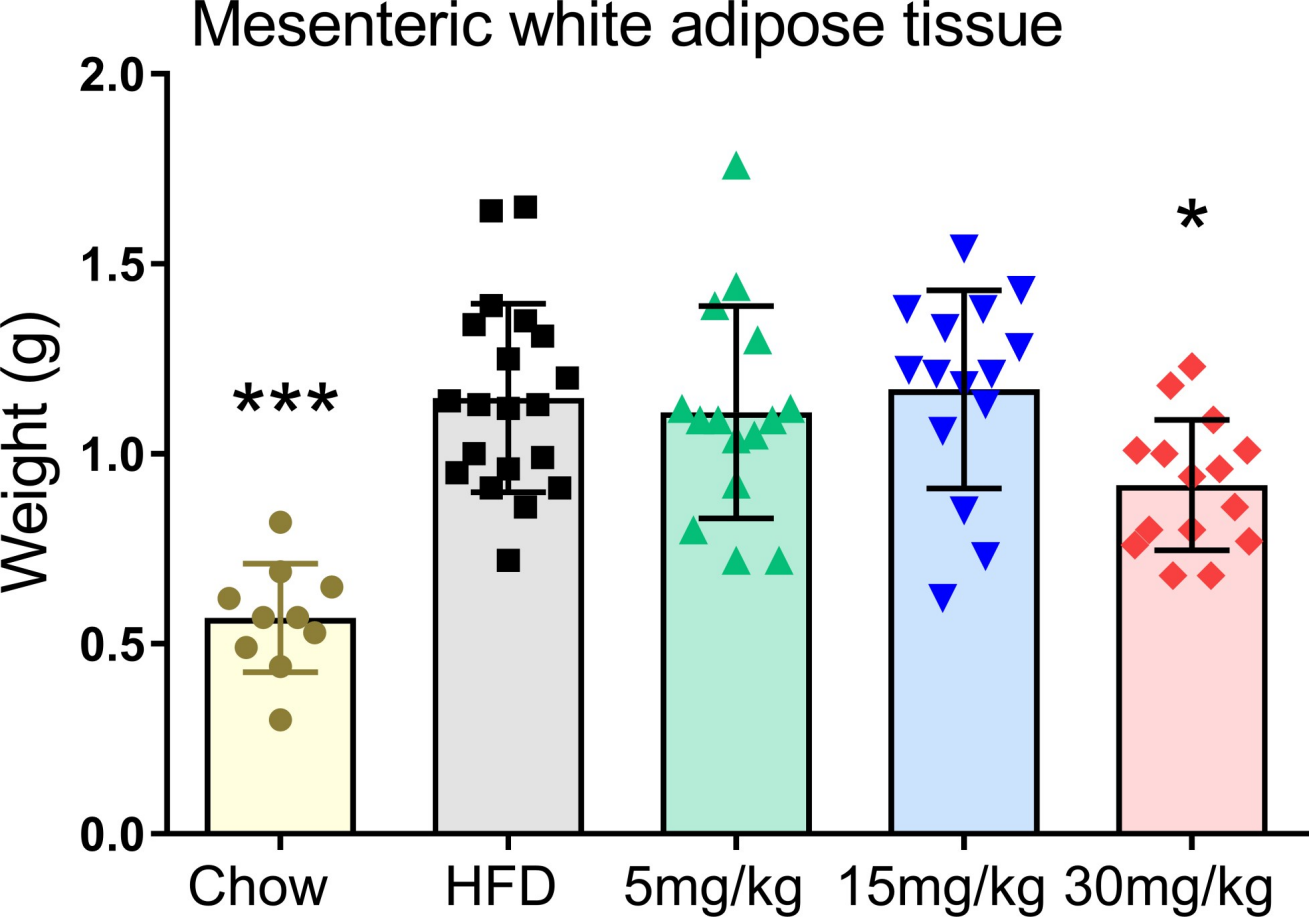
Effects of volixibat at the end of the study on mesenteric white adipose tissue weight mass. Horizontal lines indicate mean values. Error bars show standard deviation. Mesenteric white adipose tissue weight was measured after sacrifice of mice at the end of the study. *P < 0.05; ***P < 0.001 versus the HFD control group. HFD: high-fat diet. All data points shown.

In addition to the effects on the liver, the highest dose of volixibat also significantly attenuated the HFD-induced increase in mesenteric white adipose tissue weight (*P* < 0.05; Fig 8).

**Fig 9:**
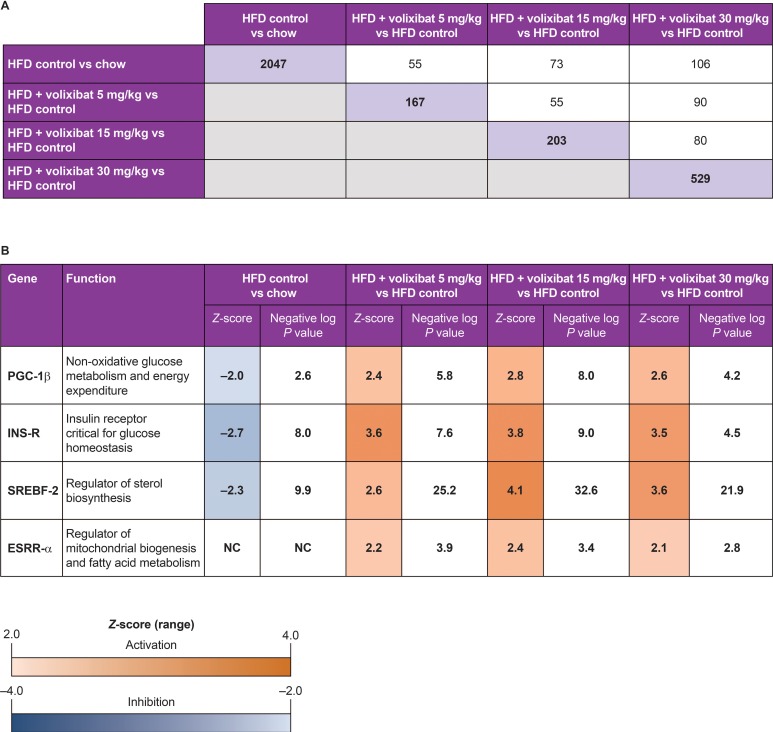
**(A) Number of differentially expressed genes by treatment; (B) differential expression of selected upstream regulators.** (A) The purple shaded boxes show the numbers of differentially expressed genes in the comparisons of chow versus HFD control groups and HFD control versus HFD + volixibat groups (each dose group). The white boxes indicate the numbers of differentially expressed genes shared between corresponding diagonal boxes (purple boxes). (B) NC indicates no consistent directional effect. The HFD control group was compared with the chow group, and each volixibat dose group was compared with the HFD control group. *P* values are shown as the negative log *P* value. The Z-score indicates the predicted direction of the effect on a transcription factor: a Z-score below –2 indicates inhibition (blue); a Z-score above +2.0 indicates activation (orange). ESRR-α: estrogen-related receptor α, HFD: high-fat diet, INS-R: insulin receptor, PGC-1β: peroxisome proliferator-activated receptor-γ coactivator-1β, SREBF-2: sterol regulatory element-binding transcription factor 2. Gene expression analysis was performed on eight mice per group.

To gain insight into the molecular processes underlying the observed hepatoprotective effects of volixibat, a more granular genome-wide gene profiling analysis was performed. Individual animals were analyzed, and the data was statistically analyzed at group level to calculate the fold changes (expressed as DEGs). A total of 2,047 hepatic genes were differentially expressed in the HFD control group compared with the chow group (Fig 9A).

The number of DEGs in the volixibat-treated groups compared with the HFD control group increased dose dependently, from 167 in the 5 mg/kg dose group up to 529 in the 30 mg/kg dose group (*P* < 0.01; Fig 9A). Forty-eight genes were differentially expressed in all volixibat treated groups (Figure A in S2 Fig), of which 32 were upregulated and 16 were downregulated. Volixibat counteracted the effect of HFD on multiple genes (Figure B in S2 Fig).

The expression of Cyp7a1 mRNA was upregulated 2.1-fold in the volixibat 5 mg/kg dose group (*P* < 0.01), 2.4-fold in the 15 mg/kg dose group (*P* < 0.01), and 1.9-fold in the 30 mg/kg dose group (*P* < 0.01). In addition, the upstream regulator analysis within the IPA software tool predicted a modest increase in Cyp7a1 activity based on the downstream target genes in all the volixibat dose groups, which is consistent with an increase in bile acid synthesis (*Z*-score 1.2–1.9, S2 Table).

The results of the upstream regulator analysis also identified four key regulators involved in energy metabolism that could account for the observed effects of volixibat on downstream gene expression: peroxisome proliferator-activated receptor-γ coactivator-1β (PGC-1β), insulin receptor (INS-R), sterol regulatory element-binding transcription factor 2 (SREBF-2), and estrogen-related receptor α (ESRR-α) (Fig 9B). Upstream regulator analysis demonstrated a marked deactivation of FXR in the HFD group (NR1H4; Z-score -2.64; *P* = 2.2E-05) than the chow group. The Z-scores for the volixibat treatment groups were -2.9 (*P* = 6.3E-08) for the 5 mg/kg, -1.5 (*P* = 3.3E-06) for the 15 mg/kg and -1.5 (*P* = 5.8E-06) for the 30 mg/kg group, indicating that only treatment with the lowest dose inactivates FXR. This upstream regulator inhibition was calculated based on the regulation of a set of more than 30 downstream FXR-target genes (S3 Fig). Furthermore, in the HFD group, the gene expression changes indicated inhibition of the upstream regulators PGC-1β, INS-R, and SREBF-2 (vs chow control; Fig 9B). Upstream pathway analysis demonstrated activation of PGC-1β, INS-R, and SREBF-2 in the volixibat groups relative to HFD, suggesting that volixibat reversed this effect. Also, activity of ESRR-α, a transcription factor implicated in mitochondrial biogenesis and fatty acid metabolism, was predicted to be significantly upregulated in the volixibat-treated mice (Fig 9B).

## Discussion

In the present study, volixibat treatment significantly attenuated the HFD-induced increase in hepatocyte hypertrophy, NAS, hepatic triglyceride and cholesteryl ester levels, and mesenteric white adipose tissue deposition. By week 24, plasma cholesterol, insulin, and ALT levels were all significantly lower in the 30 mg/kg dose group than in the HFD control group. Collectively, these findings indicate that volixibat had beneficial effects on multiple aspects of NASH pathology in this translational diet-induced NASH mouse model.[46–48]

ASBT inhibition with volixibat significantly increased total bile acid content. The observed effects are consistent with the mechanism of action of volixibat, indicating local inhibition of the ASBT in the intestinal lumen and the subsequent interruption of enterohepatic bile acid reabsorption. These effects have also been observed in clinical studies of volixibat in healthy adults,[36] overweight and obese individuals,[23] and patients with T2DM,[36] supporting the clinical relevance of this NASH mouse model. In the present study, based on gene expression and upstream regulator analysis, volixibat treatment increased Cyp7a1 expression and tended to increase Cyp7a1 activity. These changes were accompanied by significantly lower levels of hepatic cholesteryl esters and lower levels of hepatic free cholesterol, while total plasma bile acid levels remained constant. These findings support the view that volixibat can stimulate bile acid synthesis from cholesterol and can increase fecal bile acid output (i.e. the removal of bile acids from the body).

Volixibat treatment affected the fecal content of some bile acids more than others. For example, LCA and DCA fecal concentrations increased from baseline with volixibat treatment, while the fecal ω-MCA content was significantly reduced. LCA and DCA are hydrophobic, unconjugated, secondary bile acids (Fig 1) that are generally considered to be more cytotoxic and have been previously implicated in hepatocyte injury and mitochondrial dysfunction.[24] For example, treatment with cholestyramine, which increases the excretion of hydrophobic bile acids, significantly prevented development of HCC in a NASH–HCC small animal model, further supporting this hypothesis.[57] It should also be noted that, on the other hand, LCA and DCA are potent TGR5 agonists [58] and emerging evidence from mouse and human studies suggests that TGR5-mediated effects may be useful in the context of NAFLD treatment.[31]

Although volixibat did not significantly alter the total plasma bile acid level, concentrations of CDCA, DCA, TDCA, and HDCA were significantly increased, while those of CA, TCA, β-MCA, and UDCA were significantly reduced. As Fig 1 indicates, CA is conjugated with taurine in the liver and then converted to DCA by the intestinal microbiota. Plasma levels of CA and TCA were significantly lower in the volixibat groups than in the HFD control group, which is likely to be the result of blocked uptake and increased conversion into DCA in the gut. Although fecal DCA content was significantly increased with volixibat treatment, the plasma analysis suggests that a portion of DCA could have been reabsorbed in the gut, most likely by passive (ASBT-independent) transport. In addition, plasma bile acids were measured at week 22 only, so it is unclear whether this increase in plasma DCA levels is transient or sustained. It may be more likely that the levels of specific bile acids fluctuate over time depending on the availability of substrates and enzyme activity.

Changes in the levels of specific bile acids have been reported by others upon ASBT inhibition: SC-435 altered the hepatic bile acid composition towards FXR agonism in a HFD mouse model.[35] Bile acids such as CDCA, CA and DCA are reportedly FXR agonists,[59–61] although only CDCA is thought to fully activate FXR while CA and DCA act as partial agonists.[62] In the present study, plasma levels of CDCA and DCA were increased while levels of the FXR antagonist β-MCA were decreased, suggesting an overall shift towards a more FXR-agonistic bile acid pool.[35] However, upstream regulator analysis did not identify a clear activation of FXR in mouse liver tissue upon volixibat treatment (only low dose treatment was associated with FXR deactivation). One potential explanation for this could be that CDCA, which strongly activates FXR, is rapidly converted to α-MCA and β-MCA in mouse liver and that available levels are insufficient to activate FXR. An analysis of hepatic bile acids was beyond the scope of the current study which is a limitation that should be considered in future studies. Furthermore, bile acid composition is heavily influenced by a number of factors not accounted for in the present study; for example, alterations to the gut microbiota could influence the conversion of primary to secondary bile acids.[63] Comparisons between plasma and fecal bile acid species in the present study are also limited, because plasma levels were measured in individual mice whereas fecal levels were measured in each cage of two to four mice.

While the increase in total fecal bile acid content in this study was consistent with increases shown in clinical studies,[23, 36, 40] it is not certain whether the changes observed in bile acid composition in rodent models are physiologically relevant to humans. Indeed, a recent 28-day dose-escalation study of volixibat in healthy volunteers and patients with T2DM did not reveal any dose-related changes in the major serum bile acids.[44]

Mitochondrial biogenesis is impaired in NASH [64–66] and defective mitochondria are implicated in disease progression.[67] Prolonged triglyceride and free fatty acid deposition in mitochondria results in structural and molecular defects, impairing ATP synthesis and increasing oxidative stress in NASH.[67] Volixibat treatment resulted in activation of mediators of mitochondrial biogenesis, ESRR-α and PGC-1β,[68, 69] which are thought to be downregulated in T2DM.[70, 71] Activation of ESRR-α and PGC-1β by volixibat could promote mitochondrial biogenesis,[68, 69] therefore increasing respiratory and fatty acid oxidation capacity to alleviate some of the metabolic defects associated with NASH pathology. Although changes to hepatic gene expression in the HFD-fed Ldlr-/-.Leiden mouse model have been found to resemble closely those reported in patients with NASH,[47, 48] dedicated mechanistic studies are warranted to investigate the effects of volixibat on mitochondrial lipid metabolism.

Lowering blood lipid levels, for example with statins, can reduce NASH-associated liver damage.[72, 73] In the present study, volixibat was associated with significant reductions in plasma cholesterol levels. Similarly, in a clinical study of volixibat in overweight and obese adults, volixibat was associated with numerically greater reductions from baseline in total serum cholesterol level than placebo.[23] Lower plasma cholesterol levels with volixibat treatment in the present study were associated with improvements in liver pathology, including significant attenuations in NAS, hepatic triglyceride accumulation, and hepatocyte hypertrophy. Controlled long-term studies in individuals with NASH are warranted to confirm that the trends observed in this mouse model translate to a clinically relevant effect in humans.

Defective glucose metabolism and IR are key features of NASH pathophysiology.[74] INS-R activity was increased in the volixibat groups and, at the highest dose, plasma insulin levels were significantly lower than those in the HFD control group, implying an overall improvement in insulin sensitivity with volixibat treatment. Volixibat was also associated with attenuation of the HFD-induced deposition of white adipose tissue, which is also linked to IR and NASH progression.[20, 21] In individuals with NAFLD, risk factors for progression to NASH and fibrosis include diabetes, hypertension, dyslipidemia, and obesity.[51, 75]

In conclusion, this study supports the concept that ASBT inhibition may be desirable in some patients with NASH.

## Supporting information

S1 Table Overview of the statistical tests used. For normally distributed data with equal variances, a one-way analysis of variance (ANOVA) with Dunnett’s *post hoc* test was used. For data sets that were not normally distributed or did not have equal variances, a Kruskal–Wallis test was used. When the result of the Kruskal–Wallis test indicated a significant difference (*P* < 0.05), a Mann–Whitney U test was used to compare independent samples.
(DOCX)

S2 Table Analysis of transcription factors and key upstream regulators involved in major liver health categories (blue indicates inhibition; orange indicates activation). The data set shows the effects of HFD control versus chow and HFD control versus HFD + volixibat (each dose group). The significance of activation/inhibition of a given upstream regulator is indicated with the negative log *P* value (based on the Fisher’s exact test standardly used in the Ingenuity Pathway Analysis suite). NA indicates a lack of sufficient differentially expressed genes in the data set to enable linking of gene effects to an upstream regulator. A *Z*-score below –2 indicates inhibition (blue) and a *Z*-score above +2 indicates activation (orange) of the upstream regulator. HFD: high-fat diet. Gene expression analysis was performed on eight mice per group.
(DOCX)

S1 Fig Volixibat does not affect HFD-induced collagen development in the liver. A) Hepatic collagen content was determined by a biochemical analysis of hydroxyproline (an amino acid that is present in collagen) in liver tissue homogenates, B) gene expression levels of different collagens and C–F) transcriptomics-based pathway analyses showing the process ‘Early signaling events in hepatic stellate cells’ and ‘Signaling events in activated HSC’. Error bars show standard deviation. HFD: high-fat diet.
(PDF)

S2 Fig Differentially modulated genes upon volixibat treatment. A) Venn diagram showing the number of differentially expressed genes (DEGs) for each dose of volixibat, and the number of overlapping genes between the different treatment groups. In total 48 genes were modulated with all three doses. B) Heatmap showing the direction of the effect that volixibat had on these 48 genes. The effect of HFD on the expression of these genes is indicated in the left column. Red color indicates upregulation, green color downregulation.
(PDF)

S3 Fig Analysis of FXR target genes. FXR (NR1H4) and its target genes in (A) HFD vs chow group, (B) 5 mg/kg volixibat vs HFD, (C) 15 mg/kg volixibat vs HFD, (D) 30 mg/kg volixibat vs HFD. Green color indicates downregulation and red color indicates upregulation of a particular gene.
(PDF)

## Abbreviations

ALT: alanine aminotransferase
ASBT: apical sodium-dependent bile acid transporter
AST: aspartate aminotransferase
CA: cholic acid
CDCA: chenodeoxycholic acid
Cyp7a: cholesterol 17α-hydroxylase
DCA: deoxycholic acid
ESRR-αA: estrogen-related receptor α
FXR: farnesoid X receptor
HCC: hepatocellular carcinoma
HDCA: hyo-deoxycholic acid
HFD: high-fat diet
HPLC: high-performance liquid chromatography
INS-R: insulin receptor
IR: insulin resistance
LCA: lithocholic acid
MCA: muricholic acid
MS: mass spectrometry
NAFLD: non-alcoholic fatty liver disease
NAS: NAFLD activity score
NASH: non-alcoholic steatohepatitis
PGC-1βB: peroxisome proliferator-activated receptor-γ coactivator-1β
SD: standard deviation
SREBF-2: sterol regulatory element-binding transcription factor 2
T2DM: type 2 diabetes mellitus
TCA: taurine-conjugated cholic acid
TCDCA: taurine-conjugated chenodeoxycholic acid
TDCA: taurine-conjugated deoxycholic acid
UDCA: ursodeoxycholic acid
