# Supplementary material for: Apical sodium-dependent bile acid transporter inhibition with volixibat improves metabolic aspects and components of non-alcoholic steatohepatitis in Ldlr-/-.Leiden mice
Source: PLoS One. 2019 Jun 24;14(6):e0218459. doi: 10.1371/journal.pone.0218459 (PMC6590809; doi:10.1371/journal.pone.0218459)
Supplement: S1 Table — For normally distributed data with equal variances, a one-way analysis of variance (ANOVA) with Dunnett’s post hoc test was used. For data sets that were not normally distributed or did not have equal variances, a Kruskal–Wallis test was used. When the result of the Kruskal–Wallis test indicated a significant difference (P < 0.05), a Mann–Whitney U test was used to compare independent samples. (DOCX) [file pone.0218459.s001.docx]

| **Figure** | **Parameter and timepoint (weeks)** | **Statistical test used** | |
| --- | --- | --- | --- |
| 3A | Body weight  t=0  t=4, t=8, t=12, t=16, t=20 and t=24 | one-way ANOVA  one-way ANOVA | Dunnett's post hoc |
| 3B | Food intake  All time-points | one-way ANOVA |  |
| 4A | Total fecal bile acid content  t=0  t=12  t=22 | one-way ANOVA  one-way ANOVA  one-way ANOVA | Dunnett's post hoc Dunnett's post hoc |
| 4B | LCA content in feces  t=0  t=12  t=22 | one-way ANOVA  Kruskal–Wallis  Kruskal–Wallis | Mann–Whitney U test Mann–Whitney U test |
| 4C | DCA content in feces  t=0  t=12  t=22 | Kruskal–Wallis  Kruskal–Wallis  one-way ANOVA | Mann–Whitney U test Dunnett's post hoc |
| 4D | α-MCA content in feces  t=0  t=12  t=22 | Kruskal–Wallis  one-way ANOVA Kruskal–Wallis | Mann–Whitney U test  Mann–Whitney U test Mann–Whitney U test |
| 4D | α-MCA content in feces  t=0  t=12  t=22 | Kruskal–Wallis  one-way ANOVA Kruskal–Wallis | Mann–Whitney U test  Mann–Whitney U test Mann–Whitney U test |
| 4E | ω-MCA content in feces  t=0  t=12  t=22 | one-way ANOVA  Kruskal–Wallis  Kruskal–Wallis | Mann–Whitney U test Mann–Whitney U test |
| 4F | CA content in feces  t=0  t=12  t=22 | Kruskal–Wallis  one-way ANOVA  Kruskal–Wallis | Mann–Whitney U test |
| 4G | HDCA/UDCA content in feces  t=0  t=12  t=22 | Kruskal–Wallis  one-way ANOVA  one-way ANOVA | Dunnett's post hoc  Mann–Whitney U test |
| 5A-5J | Total plasma bile acids, CA, TCA, β-MCA, UDCA, DCA, TDCA, HDCA, CDCA, TCDCA | Kruskal–Wallis | Mann–Whitney U test |
| 6A | Plasma cholesterol  t=0  t=4, t=8, t=12, t=16 and t=24  t=20 | Kruskal–Wallis  Kruskal–Wallis  one-way ANOVA | Mann–Whitney U test  Dunnett's post hoc |
| 6B | Plasma insulin  t=0  t=4, t=12, t=16 t=20 and t=24 | Kruskal–Wallis  Kruskal–Wallis | Mann–Whitney U test |
| 6C | Plasma ALT  t=0  t=12 and t=24 | Kruskal–Wallis  Kruskal–Wallis | Mann–Whitney U test |
| 6D | Plasma AST  t=0  t=12  t=24 | one-way ANOVA  one-way ANOVA  Kruskal–Wallis | Dunnett's post hoc  Mann–Whitney U test |
| 6E | Plasma triglycerides  t=0, t=4, t=8 and t=16  t=12, t=20 and t=24 | Kruskal–Wallis  Kruskal–Wallis | Mann–Whitney U test |
| 6E | Plasma lipoprotein profiles | No statistical analysis was used since all the samples were pooled per group |  |
