# Supplementary figures and images for: Apical sodium-dependent bile acid transporter inhibition with volixibat improves metabolic aspects and components of non-alcoholic steatohepatitis in Ldlr-/-.Leiden mice
Source: PLoS One. 2019 Jun 24;14(6):e0218459. doi: 10.1371/journal.pone.0218459 (PMC6590809; doi:10.1371/journal.pone.0218459)

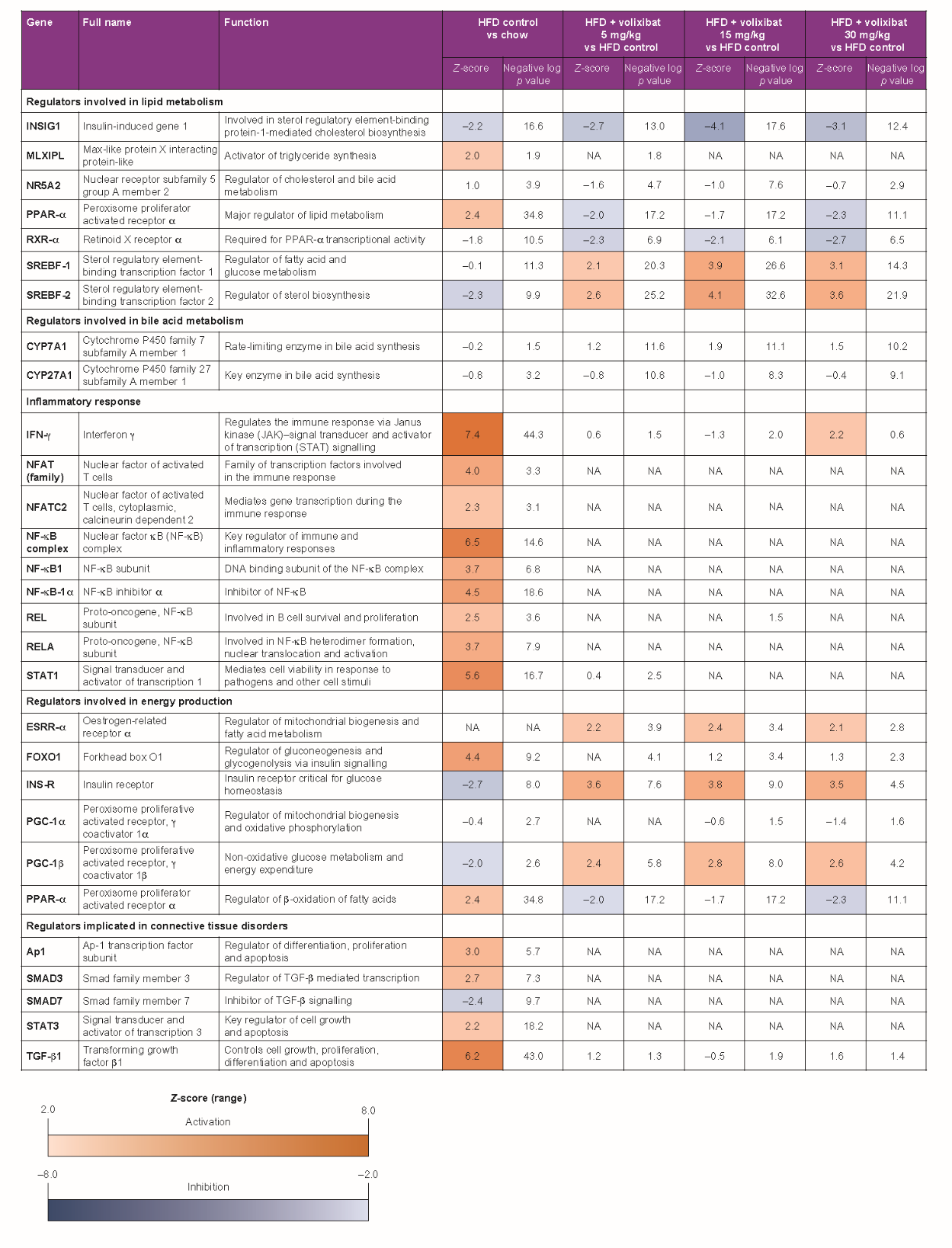

Supplement: S2 Table — The data set shows the effects of HFD control versus chow and HFD control versus HFD + volixibat (each dose group). The significance of activation/inhibition of a given upstream regulator is indicated with the negative log P value (based on the Fisher’s exact test standardly used in the Ingenuity Pathway Analysis suite). NA indicates a lack of sufficient differentially expressed genes in the data set to enable linking of gene effects to an upstream regulator. A Z-score below –2 indicates inhibition (blue) and a Z-score above +2 indicates activation (orange) of the upstream regulator. HFD: high-fat diet. Gene expression analysis was performed on eight mice per group. (DOCX) [file pone.0218459.s002.docx]

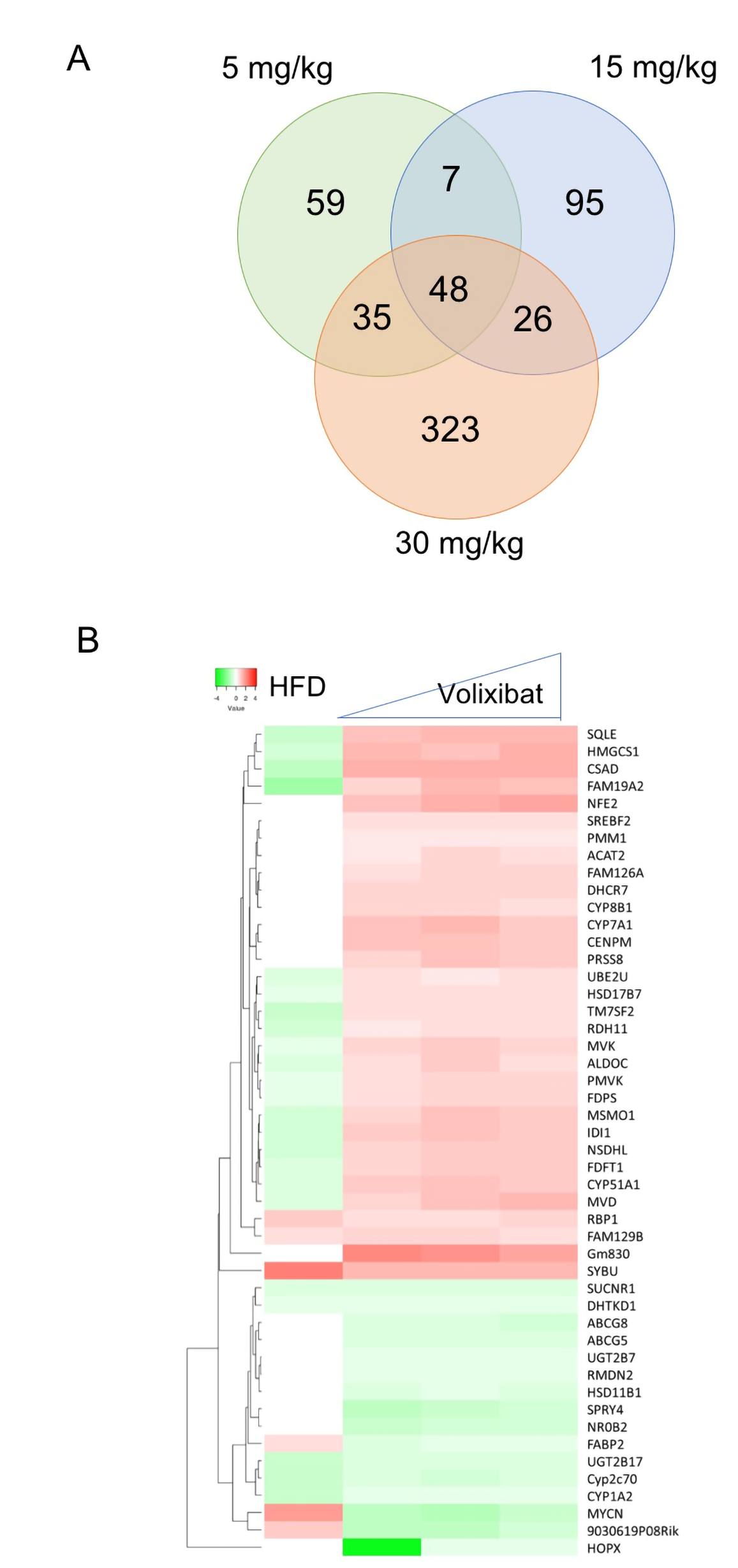

Supplement: S2 Fig — A) Venn diagram showing the number of differentially expressed genes (DEGs) for each dose of volixibat, and the number of overlapping genes between the different treatment groups. In total 48 genes were modulated with all three doses. B) Heatmap showing the direction of the effect that volixibat had on these 48 genes. The effect of HFD on the expression of these genes is indicated in the left column. Red color indicates upregulation, green color downregulation. (PDF) [file pone.0218459.s004.pdf]

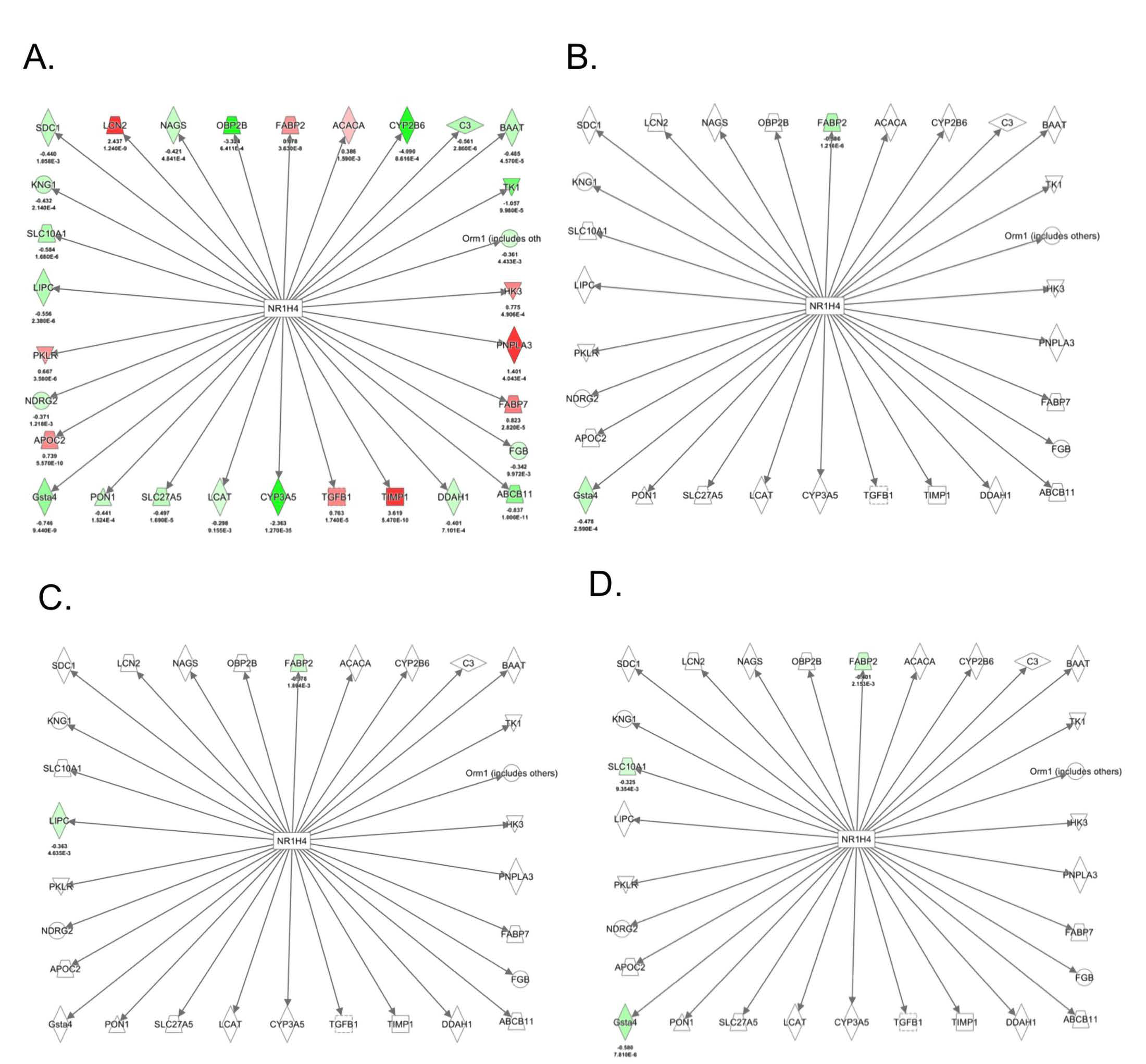

Supplement: S3 Fig — FXR (NR1H4) and its target genes in (A) HFD vs chow group, (B) 5 mg/kg volixibat vs HFD, (C) 15 mg/kg volixibat vs HFD, (D) 30 mg/kg volixibat vs HFD. Green color indicates downregulation and red color indicates upregulation of a particular gene. (PDF) [file pone.0218459.s005.pdf]
