# Supplementary material for: Apical sodium-dependent bile acid transporter inhibition with volixibat improves metabolic aspects and components of non-alcoholic steatohepatitis in Ldlr-/-.Leiden mice
Source: PLoS One. 2019 Jun 24;14(6):e0218459. doi: 10.1371/journal.pone.0218459 (PMC6590809; doi:10.1371/journal.pone.0218459)

A.

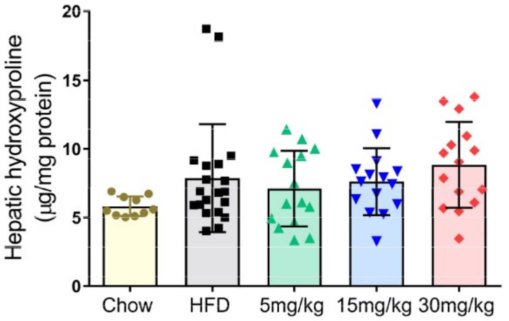

Hepatic hydroxyproline
(μg/mg protein)
20
15
10
5
0
Chow
HFD
5mg/kg
15mg/kg
30mg/kg

B.

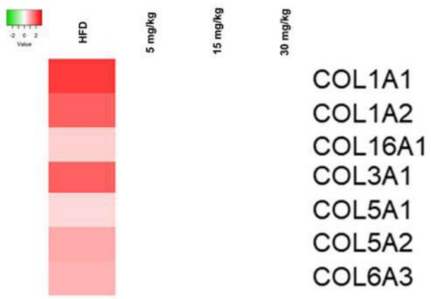

HFD
5 mg/kg
15 mg/kg
30 mg/kg
COL1A1
COL1A2
COL16A1
COL3A1
COL5A1
COL5A2
COL6A3

C.

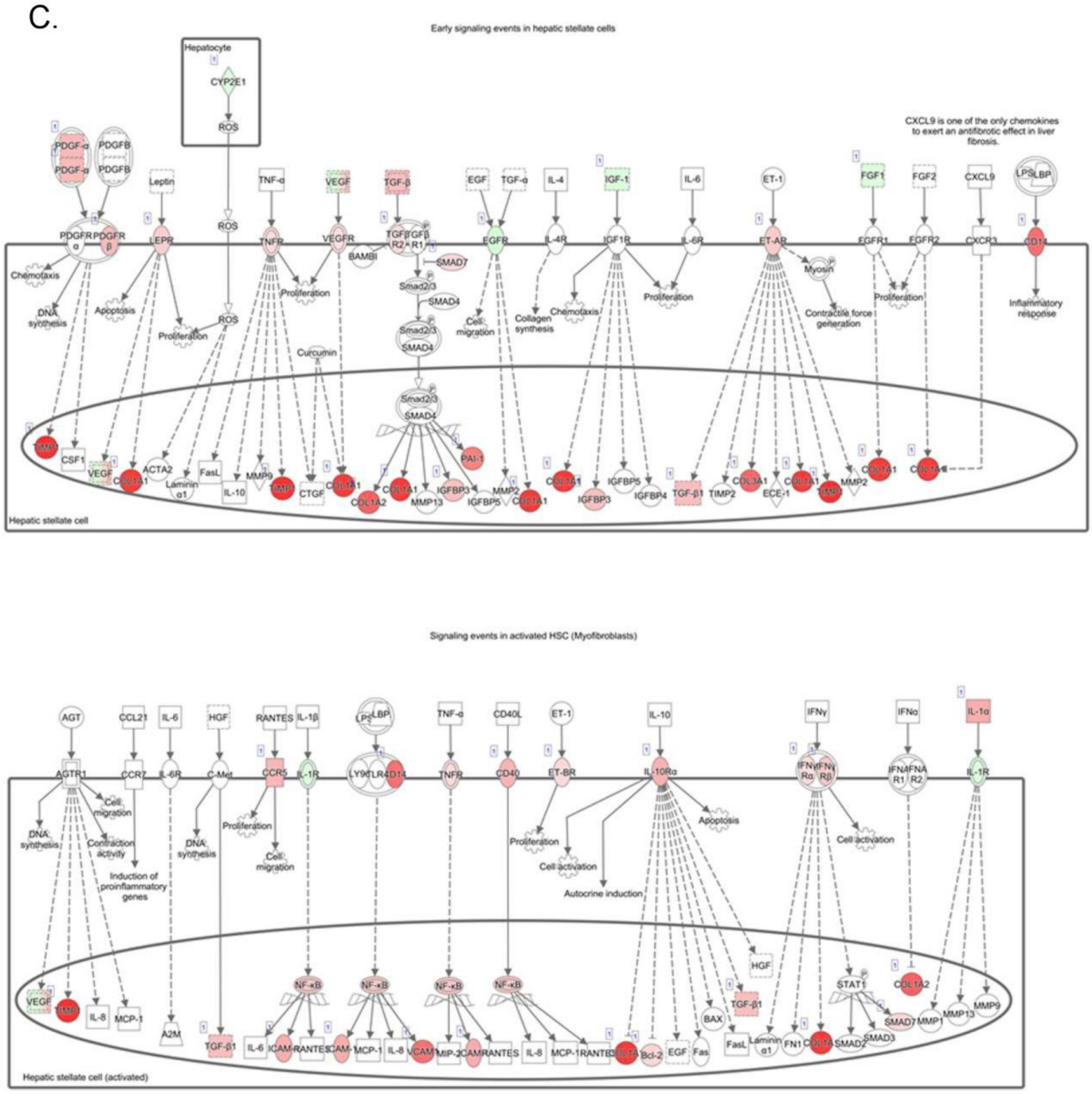

Early signaling events in hepatic stellate cells
Hepatocyte
CYP2E1
ROS
CXCL9 is one of the only chemokines to exert an antifibrotic effect in liver fibrosis.
Hepatic stellate cell
Signaling events in activated HSC (Myofibroblasts)
Hepatic stellate cell (activated)

D.

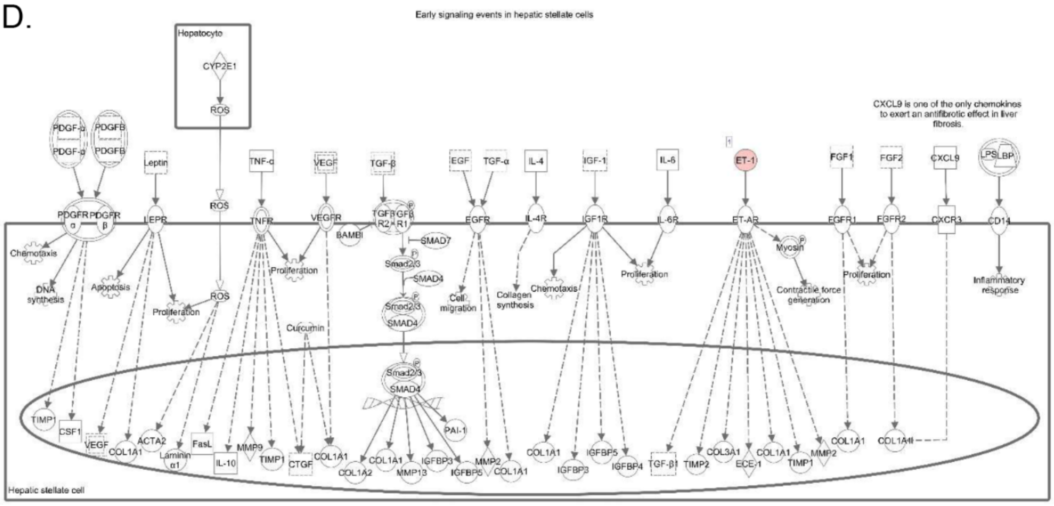
Early signaling events in hepatic stellate cells
Hepatocyte
CYP2E1
ROS
CXCL9 is one of the only chemokines to exert an antifibrotic effect in liver fibrosis.
PDGF-α
PDGFB
Leptin
TNF-α
VEGF
TGF-β
EGF
TGF-α
IL-4
IGF-1
IL-6
ET-1
FGF1
FGF2
CXCL9
LPS
LBP
PDGFR α
PDGFR β
LEPR
TNFR
VEGFR
BAMBI
SMAD7
Smad2/3
SMAD4
EGFR
IL-4R
IGF1R
IL-6R
ET-AR
Myosin
EGFR1
FGFR2
CXCR3
CD14
Chemotaxis
DNA synthesis
Apoptosis
Proliferation
Curcumin
Cell migration
Collagen synthesis
Contractile force generation
Inflammatory response
TIMP1
CSF1
COL1A1
ACTA2
Laminin α1
FasL
IL-10
MMP9
CTGF
COL1A2
MMP13
IGFBP3
IGFBP5
PAI-1
MMP2
IGFBP4
TGF-β1
TIMP2
COL3A1
ECE-1
Hepatic stellate cell

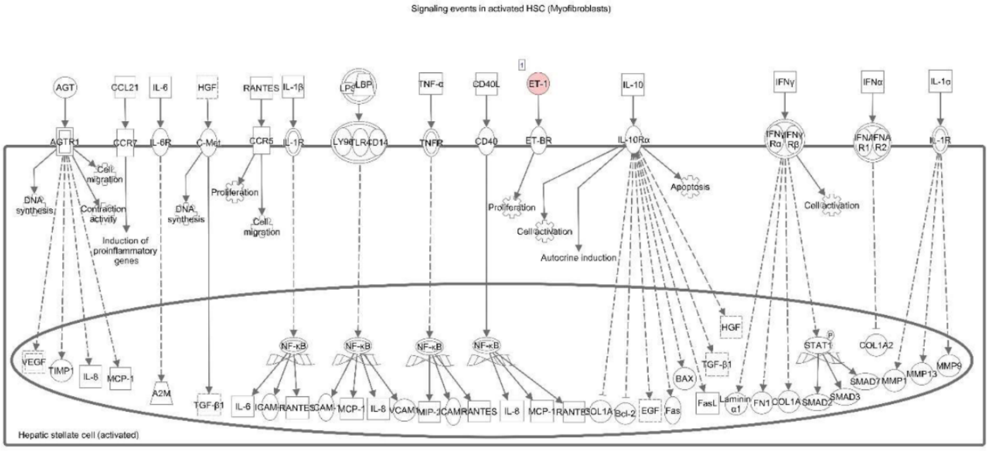
Signaling events in activated HSC (Myofibroblasts)
AGT
CCL21
IL-6
HGF
RANTES
IL-1β
LPS
LBP
TNF-α
CD40L
ET-1
IL-10
IFNγ
IFNα
IL-1α
AGTR1
CCR7
IL-6R
C-Met
CCR5
IL-1R
LY96
TLR4
CD14
TNFR
CD40
ET-BR
IL-10Rα
Cell migration
DNA synthesis
Contraction activity
Induction of proinflammatory genes
Proliferation
Cell activation
Autocrine induction
Apoptosis
NF-κB
HGF
TGF-β1
STAT1
COL1A2
VEGF
TIMP1
IL-8
MCP-1
A2M
IL-6
RANTES
VCAM1
MIP-2
BAX
Bcl-2
EGF
Fas
FasL
Laminin α1
FN1
SMAD2
SMAD3
SMAD7
MMP1
MMP13
MMP9
Hepatic stellate cell (activated)

E.

Early signaling events in hepatic stellate cells

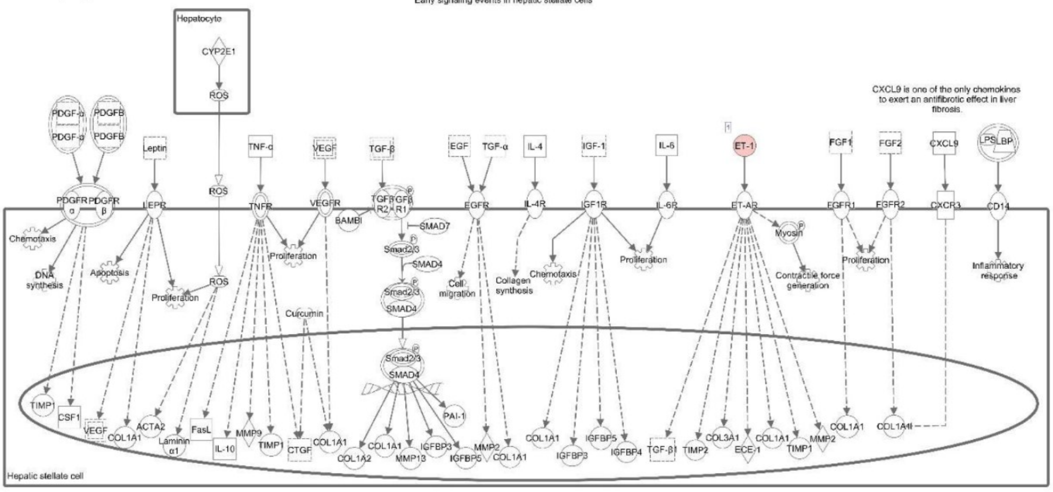

Signaling events in activated HSC (Myofibroblasts)

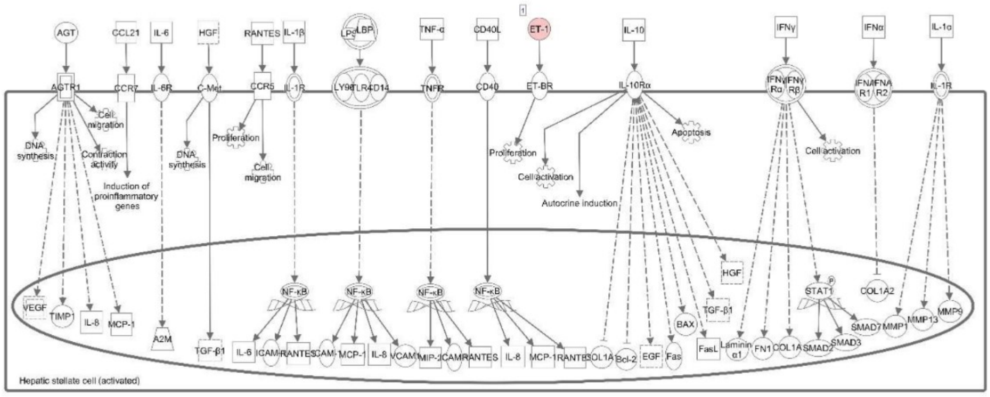

F.

Early signaling events in hepatic stellate cells

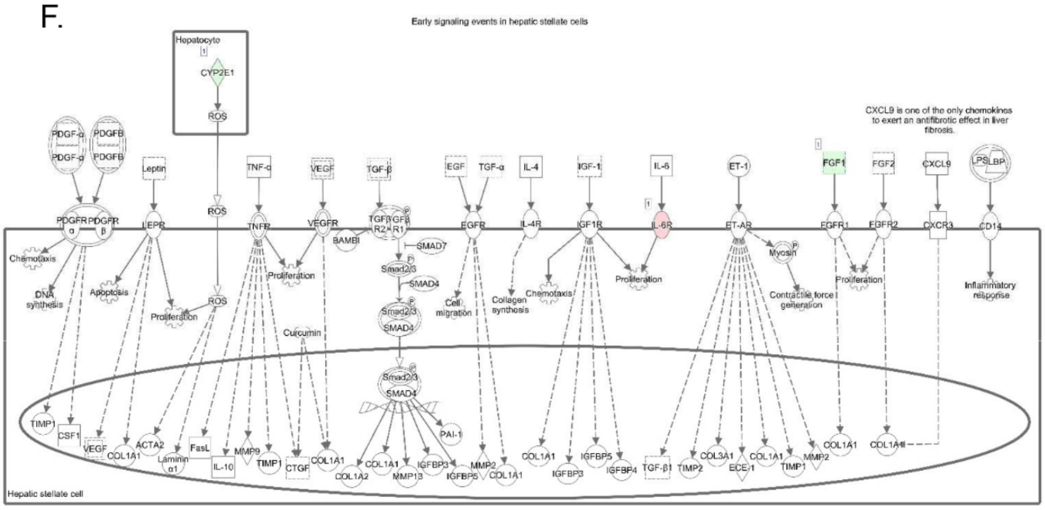

Signaling events in activated HSC (Myofibroblasts)

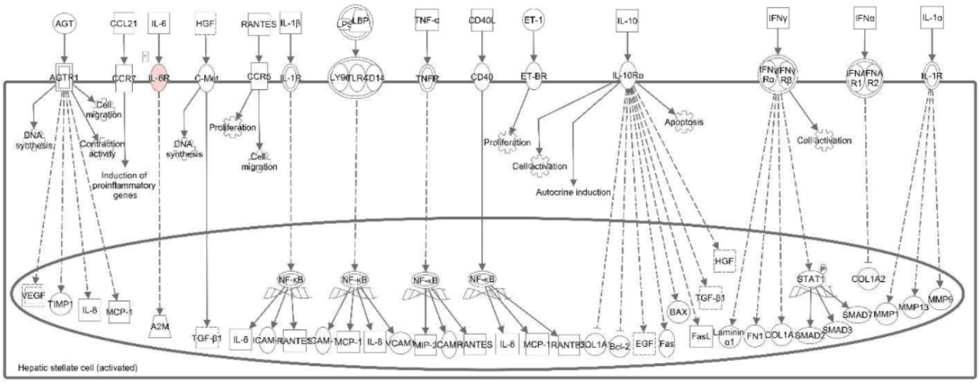

Supplement: S1 Fig — A) Hepatic collagen content was determined by a biochemical analysis of hydroxyproline (an amino acid that is present in collagen) in liver tissue homogenates, B) gene expression levels of different collagens and C–F) transcriptomics-based pathway analyses showing the process ‘Early signaling events in hepatic stellate cells’ and ‘Signaling events in activated HSC’. Error bars show standard deviation. HFD: high-fat diet. (PDF) [file pone.0218459.s003.pdf]
